# Patient reported outcome measures in intra-peritoneal adhesive disease: a scoping review

**DOI:** 10.1007/s10151-026-03339-z

**Published:** 2026-05-17

**Authors:** Reid R. Christensen, Angela M. Bailey, Aron P. Bercz, Tess C. Huy, Isabel K. Eng, Mark D. Girgis, Jason Liu, Clifford Y. Ko, Samuel P. Carmichael, Melinda Maggard Gibbons, Tara A. Russell

**Affiliations:** 1 Department of Surgery, Wake Forest University School of Medicine, Winston-Salem, NC USA; 2 Department of Surgery, Trinity Health Ann Arbor, Ypsilanti, MI USA; 3 Department of Surgery, Icahn School of Medicine at Mt. Sinai, New York, NY USA; 4 Department of Surgery, David Geffen School of Medicine at UCLA, Los Angeles, CA USA; 5 Department of Surgery, MD Anderson Cancer Center, Houston, TX USA; 6 American College of Surgeons, Chicago, IL USA

**Keywords:** Patient Reported Outcome Measures, Tissue adhesions, Quality of life, Intestinal obstruction

## Abstract

**Background:**

Intra-peritoneal adhesive disease (IPAD) can lead to debilitating symptoms including bowel obstruction, chronic abdominopelvic pain, and infertility. Standard tools to evaluate patient experiences for this disease process have not been described. This scoping review aims to (1) identify existing Patient Reported Outcome Measures (PROMs) related to IPAD and (2) evaluate the utility and quality of available IPAD-related PROMs, measured by predefined adhesive disease domains.

**Methods:**

Systematic searches were completed in PubMed using terms specific to IPAD-related symptoms and Quality of Life (QoL) domains including chronic abdominopelvic pain, bowel obstruction and function, and psychological impact on QoL. PROMs that reported exclusively on QoL were excluded. PROM development setting, psychometrics, and inclusion of adhesive disease domains were compared.

**Results:**

Of 3290 articles originally identified, 38 articles yielded 10 PROMs related to IPAD. Patient input was considered in 80% of PROM development and 60% of field testing. Reliability testing and construct validity was performed for 70% of IPAD-related PROMs. All 10 IPAD-related PROMs assessed symptoms and QoL domains (i.e. chronic abdominopelvic pain, bowel obstruction/dysfunction, and impact on QoL). No PROM specific to IPAD was identified. The Memorial Sloan Kettering Bowel Function Instrument (MSK-BFI) included the fewest (3/10) and the Small Bowel Obstruction Questionnaire (SBO-Q) covered the most IPAD-related domains (9/10).

**Conclusions:**

There is currently no IPAD-specific PROM, though the SBO-Q was identified as the most robust, as it addressed multiple symptom/QoL domains and underwent reliability/construct validity testing. Future directions include external validation of the SBO-Q in IPAD-relevant patient populations and further refinement of an IPAD-specific PROM with patient and expert input.

**Supplementary Information:**

The online version contains supplementary material available at 10.1007/s10151-026-03339-z.

## Introduction

Intra-peritoneal adhesive disease (IPAD) is a widespread and debilitating problem for patients undergoing abdominopelvic operations, conferring a life-long risk of small bowel obstruction (SBO), female infertility, chronic pain, and reoperation for surgical lysis of adhesions. In fact, 26.7% of patients are readmitted within 5 years of their index abdominopelvic operations for reasons related to IPAD (i.e. small bowel obstruction, chronic abdominal pain), and adhesiolysis accounts for more than 300,000 hospitalizations annually in the USA alone [1, 2]. While clinically available agents for adhesions prevention exist and have been associated with a decrease in adhesions formation, none have demonstrated a meaningful reduction in IPAD-related complications including small bowel obstruction [3].

Investigations into the prevention and treatment of IPAD are limited by the fact that degree of adhesions burden cannot reliably predict the impact of adhesive disease on patients. Furthermore, the range of symptoms in IPAD are often nonspecific and can overlap with other medical conditions, causing significant psychological distress and necessitating a multifactorial approach in management similar to that of other chronic GI disorders [4]. Quality of Life (QoL) in inflammatory bowel disease (IBD), for example, has been widely studied and QoL metrics are often used to evaluate the efficacy of an intervention [5, 6]. With few exceptions, the adhesions literature is limited by the paucity of studies using QoL as an end point for reduction of adhesive disease [7, 8]. Therefore, evaluating the degree of adhesions burden using metrics such as rate of hospital readmissions or adhesiolysis is missing a large portion of the IPAD population living with the chronic sequelae of this disease [9].

Researchers have made efforts to define core outcomes in the context of IPAD, and organizations such as the International Adhesions Society and the International Pelvic Pain Society provide support for patients with IPAD; however, no standardized scoring system exists to characterize the patient experience or assess their response to treatment [10]. One commonly utilized method to describe patient experience of a disease is a patient reported outcome measure (PROM), a standardized questionnaire completed by patients that provides insight into their health-related quality of life (HRQL) [11]. PROMs are increasingly being applied in clinical practice and research, allowing clinicians and researchers to measure and monitor a patient’s experience longitudinally in a standardized, consistent fashion [12]. Therefore, PROMs offer a potential tool clinicians can use to evaluate the burden of IPAD in their patients.

Given that adhesive disease symptoms often overlap with other common postoperative sequelae, we took a scoping approach to identify currently available PROMs related to the most common symptoms of adhesive disease, such as bowel obstruction or dysfunction, infertility, abdominopelvic pain, and impact on QoL and sexual function. This study aims to identify and critically evaluate previously developed PROMs related to IPAD clinical sequelae. With a PROM that effectively assesses IPAD-related symptoms and QoL, clinicians and investigators can better inform the efficacy of their treatment by accounting for patient experience in addition to clinical end points such as reduction of adhesive small bowel obstruction.

## Methods

To assess available PROMs related to IPAD, we applied a scoping review approach to identify available evidence and highlight knowledge gaps [13]. Throughout the process, the Preferred Reporting Items for Systematic Reviews and Meta-Analyses for Scoping Reviews (PRISMA-ScR) guidelines were followed [14]. The study did not constitute human subject research.

### Study design

The goal of identifying a PROM for patients with IPAD was approached by first identifying existing PROMs related to IPAD, including those specific to related symptoms/sequelae. IPAD-related outcomes included: symptoms of postoperative nausea and vomiting, obstipation or significantly diminished passage of flatus or stool, cramping or colicky abdominal pain, and abdominal bloating. Symptoms also included abdominopelvic pain persisting beyond 30 days postoperation, dietary limitations, diminished quality of life including limitations in activities of daily living (ADLs), and negative effects on mental health or sexual function. Following the identification of PROMs evaluating multiple of these domains, currently available tools were evaluated for their ability to address both IPAD-specific symptoms AND quality of life measures. Selected studies were then critically evaluated for reliability and validity.

### Adhesive disease domains

We focused on four symptomatic and QoL domains representing the most common clinical sequelae of IPAD, based on the most frequently cited complications of the disease. Symptomatic domains included (1) chronic abdominopelvic pain, (2) partial/complete bowel obstruction symptoms, and (3) bowel dysfunction (i.e. obstipation). Subdomains were created by the study team to classify specific aspects of each symptomatic and QoL domain and be inclusive of the most frequently encountered complaints shared by patients with IPAD. The chronic abdominopelvic pain domain was further divided into subdomains: (1) abdominal pain, (2) pelvic pain, and (3) abdominal bloating. Bowel obstruction subdomains included: (1) obstructive symptoms, and (2) food restriction. Bowel dysfunction did not include any subdomains. The QoL domain was further separated into subdomains: (1) general impact of adhesions on QoL, (2) psychiatric QoL, (3) impact on ADLs, and (4) sexual function.

### Literature search strategy

The MEDLINE database was searched via PubMed to identify articles involving the use or development of PROMs, in addition to terms related to our four domains of IPAD sequelae outlined above. MeSH subject headings and keywords were searched within title and abstract fields. (Supplementary Fig. S1) The following study types were included: randomized control trials, systematic reviews/meta-analyses, and observational studies. Conference abstracts, case reports, qualitative interviews, focus groups, articles without full-text ability, non-English studies, and animal studies were excluded. Studies were limited to those published between 2000 and 2024. An initial search for intraabdominal adhesive disease was performed on 2 May 2024. To ensure that our search strategy appropriately captured our entire target patient population (inclusive of patients with both abdominal and pelvic adhesions), a second search for intrapelvic adhesive disease was performed on 6 June 2024. The results of these two searches were combined, and duplicates were removed.

### Study inclusion/exclusion criteria

Studies were included if they reported on postoperative PROMs in adult patients (> 18 years of age) experiencing symptoms of bowel obstruction and/or chronic abdominopelvic pain outside of the perioperative/short-term postoperative period (> 30 days). Studies were excluded if they focused solely on the following disease states, surgical specialties, or procedures: fecal or urinary incontinence, perianal disease, malignancy, pelvic organ prolapse, transplant surgery, strictureplasty, stoma, ileal pouch anal-anastomosis, or low anterior resection. PROMs that reported exclusively on symptoms or quality of life outside of predefined adhesive disease domains were excluded. Additionally, PROMs that evaluated only IPAD-related symptoms without QoL, or only QoL without symptoms, were excluded as our aim was to identify a PROM that comprehensively evaluated symptoms and QoL in the same clinical tool. However, PROMs that were excluded on this basis were still evaluated by the study team for background information and are summarized in Supplementary Table S1. PROMs meeting final inclusion criteria addressed at least one of the predefined adhesive disease symptom domains and quality of life (hereafter referred to as IPAD-related PROMs).

### Study selection

Title and abstract screening were conducted by eight authors (R.R.C., A.M.B., A.P.B., T.C.H., I.K.E., S.P.C., M.M.G., T.A.R.). Articles that did not meet inclusion criteria after title and abstract screening were excluded. Full-text articles were then retrieved and assessed according to the inclusion/exclusion criteria. PROMs were extracted from reviewed articles and assessed for inclusion. Each article was dual screened by the authors, with disagreements resolved by a third author.

### Data extraction/content analysis

The general characteristics of each PROM that met inclusion criteria were assessed, including the number of questions/items, the setting of development (i.e. year and country), and the presence or absence of patient input in development and field testing. Each PROM’s reliability was assessed by the original study’s report of internal consistency (the extent to which items in a scale measure the same concept, measured with Cronbach’s alpha) and test–retest reliability (the extent to which consistent results are produced when scales are administered at different time points, measured by intraclass coefficient (ICC)). Greater than 0.7 was considered acceptable for both Cronbach’s alpha and ICC. Each PROM was also assessed for construct validity, specifically whether each tool was evaluated for convergent validity and discriminant validity. Each PROM was assessed for whether the tool had convergent > discriminant validity, as a measure for degree of overall psychometric validity. Finally, the content of each PROM was analyzed and characterized by the extent to which their items (questions) addressed the adhesive disease symptom and QoL domains of interest.

## Results

### Identification of IPAD-related PROMs

**Fig. 1:**
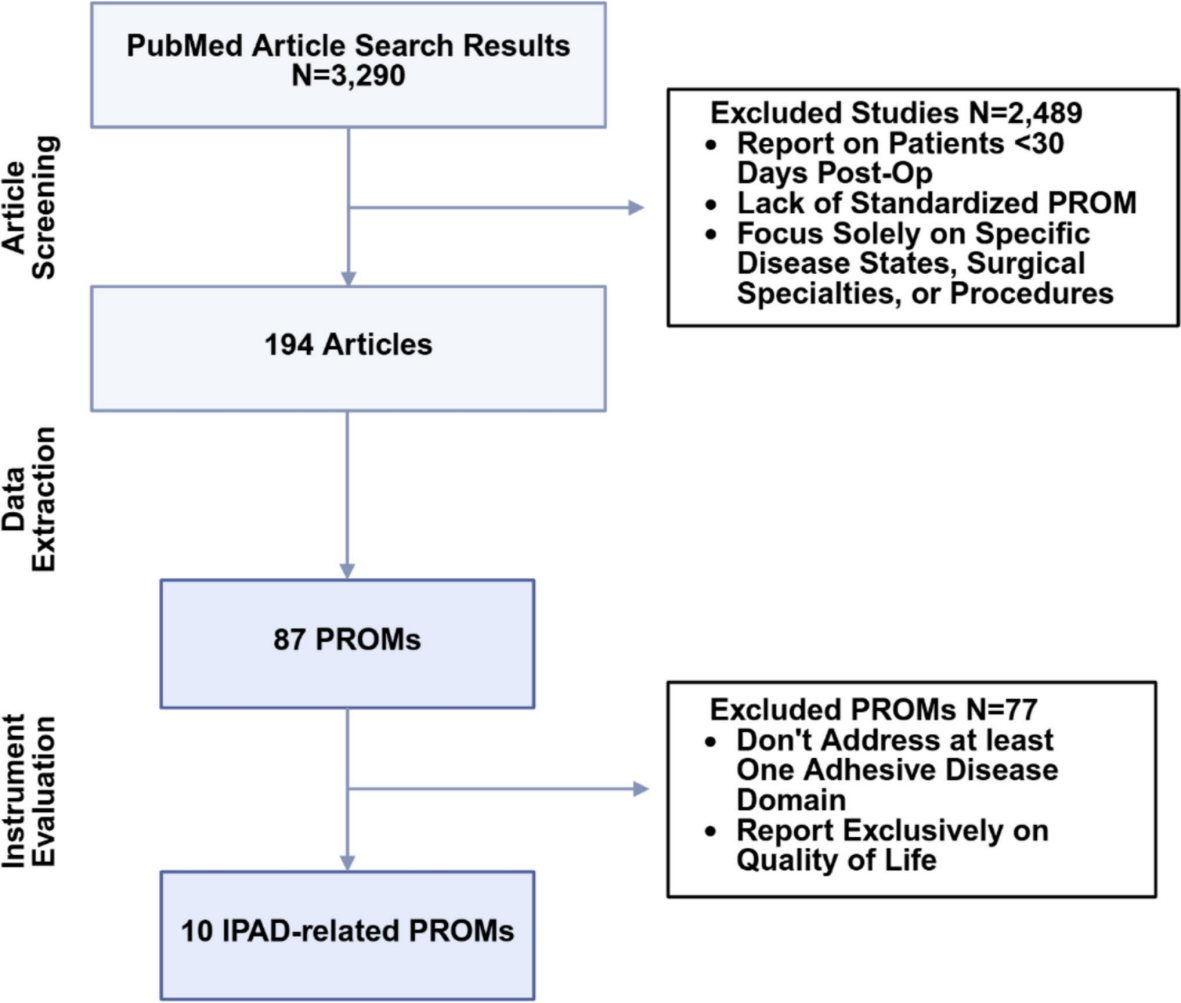
CONSORT diagram detailing PubMed search results and process of identifying IPAD-related PROMs

A total of 3290 articles were identified by our literature search. Of these, 2489 articles were excluded for the following reasons: reporting on patients in the early postoperative period (< 30 days), lack of standardization not representing a true PROM, and focusing on disease states, surgical specialties, or procedures that were not specific to IPAD. Of the remaining articles, 87 PROMs were identified, and 10 met the criteria for an IPAD-related PROM. The remaining 77 PROMs were excluded as they did not address at least one of the adhesive disease symptom domains. (Fig. 1).

### Development setting

The setting in which IPAD-related PROMs were developed are summarized in Table 1. PROMs were developed between 1989 and 2014 across North American and European countries in the following disease states: colorectal cancer, inflammatory bowel disease, benign gastrointestinal disease, and gynecologic disease. Patient input was considered in 80% (*n* = 8/10) of PROM development and 60% (*n* = 6/10) of PROM field testing.

**Table 1:** Setting of PROM development and presence/absence of patient input in initial development and field testing among IPAD-related PROMs

Patient Reported Outcome Measure (PROM)	Year, country of development	Patient input in PROM		# Questions
		Development	Field testing	
Colorectal cancer				
Colorectal Functional Outcome (COREFO)	2005, Netherlands	✓	✓	26
EORTC* Quality of Life Colorectal Cancer Module (QLQ-CR29)	2007, UK	✓	✓	29
EORTC* Quality of Life Colorectal Cancer Module (QLQ-CR38)	1999, Netherlands	–	✓	38
Memorial Bowel Function Instrument Urgency Subscale	2004, USA	✓	✓	18
Inflammatory bowel disease				
Inflammatory Bowel Disease Questionnaire (IBDQ)	1989, Canada	✓	–	32
Padova Inflammatory Bowel Disease Quality of Life Survey	1995, Italy	–	–	29
Short Inflammatory Bowel Disease Questionnaire (SIBDQ)	1996, Canada + USA	✓	–	10
Benign gastroenterology				
Gastrointestinal Quality of Life Index (GIQLI)	1995, UK & Germany	✓	–	36
Small Bowel Obstruction Questionnaire (SBO-Q)	2014, USA	✓	✓	36
Gynecologic				
Uterine Fibroid Symptom Score (UFS QoL)	2002, USA	✓	✓	37

*EORTC* European Organization for Research and Treatment of Cancer Quality of Life

### Psychometric evaluation

Psychometrics of IPAD-related PROMs are summarized in Table 2. Reliability testing was performed for 70% of PROMs. Approximately 50% reported Cronbach’s alpha (internal reliability), and 70% reported test–retest reliability. The internal reliability varied widely across PROMs. The test–retest reliability was more consistent as all PROMs that reported this metric had a test–retest reliability > 0.7. Approximately 70% (*n* = 7/10) of PROMs reported on construct validity, defined as convergent and/or discriminant validity, with four PROMs reporting on both convergent and discriminant validity. Only one study, the COREFO, demonstrated a convergent validity that was greater than discriminant validity, although this was only the case for two of its five scales [15].

**Table 2:** IPAD-related PROM Psychometrics including number of questions, reliability, and construct validity. Scale represents a grouping of questions that measures a particular construct *e.g.* incontinence

Patient Reported Outcome Measure	# Scales	Reliability		Construct Validity		
		Internal reliability (Cronbach’s Alpha)	Test–retest reliability (ICC*)	Convergent validity tested	Discriminant validity tested	Convergent > discriminant validity
Colorectal Functional Outcome (COREFO)	5	3/5 scales > 0.7	5/5 scales > 0.7	✓	✓	For 2/5 scales
EORTC* Quality of Life Colorectal Cancer Module (QLQ-CR29)	6	–	–	–	–	–
EORTC* Quality of Life Colorectal Cancer Module (QLQ-CR38)	9	7/9 scales > 0.7	9/9 scales > 0.78	–	✓	–
Memorial Bowel Function Instrument Urgency Subscale	3	–	3/3 scales > 0.74	✓	✓	✕
Inflammatory Bowel Disease Questionnaire (IBDQ)	4	–	–	✓	–	–
Padova Inflammatory Bowel Disease Quality of Life Survey	4	–	–	–	–	–
Short Inflammatory Bowel Disease Questionnaire (SIBDQ)	4	All Scales > 0.7	All Scales < 0.7	✓	✓	✕
Gastrointestinal Quality of Life Index (GIQLI)	5	–	Overall: > 0.9	–	–	–
Small Bowel Obstruction Questionnaire (SBO-Q)	9	Overall: 0.86	Overall: 0.93	–	✓	–
Uterine Fibroid Symptom Score (UFS QoL)	8	All Scales > 0.7	All scales > 0.76	✓	✓	✕

*EORTC* European Organization for Research and Treatment of Cancer Quality of Life, *ICC* intraclass coefficient

### Symptoms and QoL domains

**Fig. 2:**
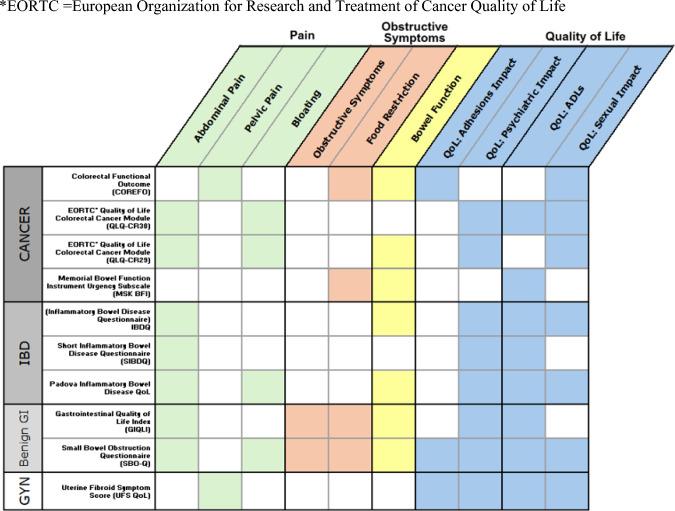
Symptom and quality of life domains across IPAD-related PROMs. Shaded boxes indicate that the PROM included items in that domain. Symptom domains are represented by green (pain), orange (obstructive symptoms), yellow (bowel function), and blue (quality of life). *EORTC* European Organization for Research and Treatment of Cancer Quality of Life

All 10 IPAD-related PROMs included items in at least one symptom domain and one QoL, as illustrated in Fig. 2. The number of questions ranged from 10 in the Short Inflammatory Bowel Disease Questionnaire (SIBDQ) to 38 in the Colorectal Cancer-Specific Quality of Life Questionnaire (QLQ-CR38) with a median of 30.5 questions [13, 16]. Among symptom domains, the Small Bowel Obstruction Questionnaire (SBO-Q) included items covering the most subdomains (*n* = 5/6) while the UFS-QoL covered the fewest subdomains (*n* = 1/6) [17, 18]. Among the QoL domains, the SBO-Q and UFS-QoL included questions in all four QoL subdomains while the Memorial Sloan Kettering Bowel Function Instrument (MSK-BFI) included questions in only one QoL subdomain [17–19]. Overall, the MSK-BFI included items in the fewest subdomains (*n* = 3/10), while the SBO-Q included items that addressed the most domains (*n* = 9/10) [18, 19].

## Discussion

This scoping review sought to identify PROMs related to IPAD and evaluate their utility as clinical assessment tools. Implementation of a PROM specific to IPAD would improve clinical assessment of disease burden and enhance future research efforts on the impact of preventative interventions.

### Key findings

While no single PROM was specifically developed to evaluate the sequela of adhesive disease, 10 PROMs were identified that included questions addressing at least one symptomatic and one QoL domain. These 10 PROMs were identified across colorectal cancer, inflammatory bowel disease, benign gastroenterology, and gynecologic fields. Of these, the Small Bowel Obstruction Questionnaire (SBO-Q) included items across most of the symptomatic and QoL subdomains (*n* = 9/10) but did not include any items in the pelvic pain subdomain.

### Psychometric evaluation

Regarding psychometrics, most studies performed reliability and construct validity testing with acceptable results in a fraction of categories. However, the internal reliability varied widely across PROMs. For example, all scales (i.e. sections of questions that measure a particular construct such as incontinence) of the Uterine Fibroid Symptom and Quality of Life Questionnaire (UFS-QoL) had a Cronbach’s alpha > 0.7 but only three of the five scales of the Colorectal Functional Outcome Questionnaire (COREFO) had a Cronbach’s alpha > 0.7 [15, 17]. None of the PROMs had promising results in both reliability and construct validity and only one, the Colorectal Functional Outcome Questionnaire (COREFO), demonstrated convergent validity greater than discriminant validity in some of its scales [15]. In other words, the COREFO was the only PROM that measured its underlying construct against comparable measures with acceptable results, emphasizing the need for further study of these PROMs before widespread dissemination in clinical practice.

### Expert consensus

Preliminary findings from this study were presented at the American College of Surgeons Adhesions Improvement Project Summit in 2024, during which a multidisciplinary group of experts, industry stakeholders, and funding agencies assembled to address the ongoing challenges of preventing and treating IPAD [20]. At this meeting, the SBO-Q was felt to represent the best IPAD-related PROM to date, given its coverage of 9/10 symptoms and QoL subdomains. The SBO-Q was also developed on the basis of patient feedback and was validated in a control population (i.e. individuals without bowel obstruction) by the researchers who developed the tool. However, its practical implementation was felt to be limited given its overall length (36 questions versus median of 30.5) and lack of specificity (i.e. differentiating between various causes of bowel obstruction). Additionally, the SBO-Q has not been validated by other researchers and has not undergone convergent validity testing, limiting our ability to assess how the tool performs relative to other IPAD-related PROMs.

### Limitations

The primary limitation of this study is the overlapping symptomatology between IPAD and various other disease states. Peritoneal adhesions pose a challenge to researchers and clinicians as they are unpredictable where they will develop within the abdomen/pelvis and whether they will be clinically significant [3]. The inconsistency in clinical manifestations can make diagnosis of IPAD difficult and may have limited our ability to capture all the symptoms and quality of life subdomains which represent IPAD. Another potential limitation inherent to our scoping review methodology is the restriction of article types and exclusion of non-English studies. This was done to ensure we were able to comprehensively review and evaluate all studies in our search based on our inclusion and exclusion criteria.

### Future directions

Future directions include: (a) determining if the SBO-Q could be used in the clinical and research setting more broadly, (b) assess if the SBO-Q could be adapted to address both abdominal *and* pelvic disease, (c) pilot the use of the SBO-Q as a tool to assess longitudinal symptoms, and (d) engaging patients to further refine SBO-Q for the IPAD patient population. Patient and provider input will be especially valuable to ensure this tool appropriately captures the relevant symptoms and QoL domains specific to IPAD. Based on our findings and expert discussion at the Adhesions Improvement Project Summit in 2024, the SBO-Q is a promising starting point for an IPAD-specific PROM. Implementation of the SBO-Q, or future iterations of a PROM similar to the SBO-Q, could be implemented into clinical practice to assess the burden of disease for patients with IPAD, as well as in a research setting measuring the efficacy of an intervention for the prevention and/or treatment of IPAD. A patient focused approach that aims at reducing all the clinical sequelae of adhesive disease, rather than a singular focus on rates of reoperation for SBO, will meaningfully assess quality and quantity of time out of the hospital.

## Conclusions

Intraperitoneal adhesive disease (IPAD) can have a lifelong, debilitating impact on patients, yet its effects on quality of life remain understudied. Although no IPAD-specific PROM exists, this work identified the SBO-Q as a promising tool for the assessment of many IPAD-related symptom and quality of life domains. For the advancement of IPAD research, it is critical that we place emphasis on the patient voice by incorporating tools including the SBO-Q into future studies and further invest in developing and refining these tools.

## Supplementary Information

Below is the link to the electronic supplementary material.

Supplementary file1 (PDF 711 KB)

## Data Availability

No datasets were generated or analyzed during the current study.
