# Supplementary material for: Patient reported outcome measures in intra-peritoneal adhesive disease: a scoping review
Source: Tech Coloproctol. 2026 May 17;30(1):115. doi: 10.1007/s10151-026-03339-z (PMC13364778; doi:10.1007/s10151-026-03339-z)
Supplement: Supplementary file 1 — Supplementary file1 (PDF 711 KB) [file 10151_2026_3339_MOESM1_ESM.pdf]

Techniques in Coloproctology, Review

Patient Reported Outcome Measures in Intra-Peritoneal Adhesive Disease: A Scoping Review

Reid R Christensen, BS[1]; Angela M Bailey, MD, MS[2]; Aron P Bercz, MD[3]; Tess C Huy, MD[4]; Isabel K Eng, MD[4]; Mark D Girgis, MD[4]; Jason Liu, MD, MS[5]; Clifford Y Ko MD, MS, MSHS[4,6]; Samuel P Carmichael, MD, PhD[1]; Melinda Maggard Gibbons, MD, MSHS[4]; Tara A Russell MD, MPH, PhD[4]

Author Addresses

[1] Wake Forest University School of Medicine, Department of Surgery, Winston-Salem, NC, USA

[2] Trinity Health Ann Arbor, Department of Surgery, Ypsilanti, MI, USA

[3] Icahn School of Medicine at Mt. Sinai, Department of Surgery, New York, NY, USA

[4] David Geffen School of Medicine at UCLA, Department of Surgery, Los Angeles, CA, USA

[5] MD Anderson Cancer Center, Department of Surgery, Houston, TX, USA

[6] American College of Surgeons, Chicago, IL, USA

Corresponding Author: Tara A Russell, 1100 Glendon Ave, Suite 810, Los Angeles, CA 90095, (310) 267-3318, trussell@mednet.ucla.edu

Supplementary Figure S1: Search strategy to identify PROMs related to IPAD. Two searches were completed – one for intra-abdominal adhesions and another for intra-pelvic adhesions.

Intra-Abdominal Adhesions Search Terms

"patient reported outcome*"[Title/Abstract] OR "patient-reported*"[Title/Abstract] OR "patient reported experience*"[Title/Abstract] OR "patient reported experience measure*"[Title/Abstract] OR "Patient Reported Outcome Measures"[MeSH Terms] OR "survey*"[Title/Abstract] OR "questionnaire*"[Title/Abstract] OR "PROM"[Title/Abstract] OR "PROMIS"[Title/Abstract] OR "PREM"[Title/Abstract]

AND

"intestinal obstruction"[mesh] OR "bowel obstruction*"[tiab] OR "intestinal obstruction*"[tiab] OR "intestine obstruction*"[tiab] OR "gastrointestinal obstruction*"[tiab] OR SBO[tiab] OR "abdominal pain"[tiab] OR intestinal disease/surgery[mesh] OR abdomen/surgery[mesh]

NOT

"endometriosis" OR "cystitis" OR "prostatitis" OR "malignant" OR "malignancy" OR cancer OR ortho* OR pregnan* OR child* OR pediatric OR paediatric OR constipation OR gastroparesis

Intra-Pelvic Adhesions Search Terms

("patient reported outcome*"[Title/Abstract] OR "patient-reported*"[Title/Abstract] OR "patient reported experience*"[Title/Abstract] OR "patient reported experience measure*"[Title/Abstract] OR "Patient Reported Outcome Measures"[MeSH Terms] OR "survey*"[Title/Abstract] OR "questionnaire*"[Title/Abstract] OR "PROM"[Title/Abstract] OR "PROMIS"[Title/Abstract] OR "PREM"[Title/Abstract]

AND

"Uterine Myomectomy"[Mesh]) OR "Hysterectomy"[Mesh] OR "Pelvic Pain/surgery"[Mesh]

NOT

"endometriosis" OR "cystitis" OR "prostatitis" OR "malignant" OR "malignancy" OR cancer OR ortho* OR pregnan* OR child* OR pediatric OR paediatric OR constipation OR gastroparesis

Supplementary Table S1: PROMs identified during data extraction that did not meet inclusion criteria. Type of PROM represents the domain (specific symptoms or general quality of life) which each tool evaluates.

CRS = Colorectal Surgery GS/EGS = General Surgery/Emergency General Surgery
GYN = Gynecology General/QoL = General/Quality of Life

Patient Reported Outcome Measure	PROM Type:	Specialties Represented
Work Productivity (WPAI)	General/QoL	CRS
Sleep Quality (PSQI)	General/QoL	CRS
SF-36	General/QoL	CRS, GS/EGS, GYN
EuroQol	General/QoL	CRS
SF-12	General/QoL	CRS, GYN
Health Status Questionnaire (HSQ)	General/QoL	CRS
WHO-BREF	General/QoL	CRS
15D	General/QoL	CRS
Giessen Sx List (GBB-24)	General/QoL	CRS
CCS	General/QoL	CRS
EQ-5D-5L	General/QoL	GS/EGS, GYN
Patient Experience of Surgery Questionnaire (PESQ)	General/QoL	GS/EGS

Duke Activity Status Index (DASI)	General/QoL	GS/EGS
EORTC QLQ-C30	General/QoL	GS/EGS
MOS-SSS (Social Support)	General/QoL	GYN
PSQI (Sleep Quality)	General/QoL	GYN
GSDS (Sleep)	General/QoL	GYN
PROMIS-PF (Physical Function)	General/QoL	GYN
WHO-BREF	General/QoL	GYN
McGill Pain Questionnaire (MPQ)	Pain	GS/EGS
S-LANSS	Pain	GS/EGS
Visual Analogue Scale (VAS)	Pain	GS/EGS
McGill Pain Questionnaire (MPQ)	Pain	GYN
DN4 (Neuropathic)	Pain	GYN
Pain Catastrophizing Scale (PCS)	Pain	GYN
Brief Pain Inventory (BPI)	Pain	GYN
Fatigue (FACIT-F)	Psych	CRS
Hospital Anxiety & Depression (HADS)	Psych	CRS
Psychological General Well Being Index (PGWBI)	Psych	CRS
Ways of coping	Psych	CRS
SSC (psychosomatic complaints)	Psych	GS/EGS
SLC-90	Psych	GS/EGS
Audit-C	Psych	GS/EGS
AAS	Psych	GS/EGS
Coping Strategies Questionnaire	Psych	GYN
Beck Depression Inventory (BPI)	Psych	GYN
Brief Symptom Inventory	Psych	GYN
Body Esteem Scale	Psych	GYN
CES-D (depression)	Psych	GYN
W-BQ energy	Psych	GYN
W-BQ positive well-being	Psych	GYN
Psych general well-being index	Psych	GYN
The Body Image Scale	Psych	GYN
Spielberger State-Trait Anxiety Inventory	Psych	GYN
